# Supplementary material for: The impact of classroom interaction on willingness to communicate: The mediating roles of speaking self-efficacy and foreign language enjoyment
Source: PLoS One. 2025 Jul 16;20(7):e0328226. doi: 10.1371/journal.pone.0328226 (PMC12266430; doi:10.1371/journal.pone.0328226)
Supplement: S1 Appendix — The complete 44 items in the questionnaire. (DOCX) [file pone.0328226.s001.docx]

**The Complete 44 Items in the Questionnaire**

**Classroom Interaction Scale**

1. I often state my opinions to the instructor during the lecture.

2. I often ask the instructor questions during the lecture.

3. I often answer or respond to questions or requests from the instructor during the

lecture.

4. I think the interaction between the instructor and me is high during the lecture.

5. The instructor often offers opinions to us during the lecture.

6. The instructor often asks us questions during the lecture.

7. The instructor often answers our questions raised during the lecture.

8. I often ask other students questions during the lecture.

9. I think there is much interaction between other students and me during the lecture.

10. I often state my opinions to other students during the lecture.

11. I often answer questions from other students during the lecture.

**Willingness to Communicate in English Scale**

1. I am willing to do a role-play standing in front of the class in English (e.g.,

ordering food in a restaurant).

13. I am willing to give a short self-introduction without notes in English to the class.

14. I am willing to give a short speech in English to the class about my hometown

with notes.

1. I am willing to translate a spoken utterance from Chinese into English in my

group.

16. I am willing to ask the teacher in English to repeat what he/she just said in English

because I didn’t understand.

17. I am willing to do a role-play in English at my desk, with my peer (e.g., ordering

food in a restaurant).

1. I am willing to ask my peer sitting next to me in English the meaning of an

English word.

1. I am willing to ask my group mates in English the meaning of a word I do not

know.

1. I am willing to ask my group mates in English how to pronounce a word in

English.

1. I am willing to ask my peer sitting next to me in English how to say an English

phrase to express the thoughts in my mind.

**Speaking Self-efficacy Scale**

1. When speaking English in the classroom, I can speak fluently.
2. When speaking English in the classroom, I can logically organize my words.
3. When speaking English in the classroom, I can speak with few pause or filler

(i.e., “Um,” “Ah,” or “You Know”).

25. When speaking English in the classroom, I can speak with grammatical accuracy.

26. When speaking English in the classroom, I can speak with correct pronunciation, intonation, and liaison.

27. I actively participate in my speaking course to improve my speaking.

28. When speaking English in the classroom, I can think of my goals before speaking.

29. When speaking English in the classroom, I can evaluate whether I achieve my goal in speaking.

30. When speaking English in the classroom, I can speak with confidence.

31. I am not stressed out when speaking English in the classroom.

32. I can understand the most difficult material presented in speaking course.

33. I can do an excellent job on the assignments and tests in the speaking course.

34. Considering the difficulty of the speaking course, the teacher, and my skill, I think I can do well in this class.

35. I can receive an excellent grade in speaking course.

**Foreign Language Enjoyment Scale**

1. I enjoy it.
2. I’ve learnt interesting things.
3. In class, I feel proud of my accomplishments.
4. The teacher is encouraging.
5. The teacher is friendly.
6. The teacher is supportive.
7. We form a tight group.
8. We have common “legends”, such as running jokes.
9. We laugh a lot.
