# Supplementary material for: The impact of classroom interaction on willingness to communicate: The mediating roles of speaking self-efficacy and foreign language enjoyment
Source: PLoS One. 2025 Jul 16;20(7):e0328226. doi: 10.1371/journal.pone.0328226 (PMC12266430; doi:10.1371/journal.pone.0328226)
Supplement: Attachment — Submitted filename: Peer Review Report_Bosheng Jing.docx [file pone.0328226.s003.docx]

**Peer Review Report**

**Manuscript Title**: *The Impact of Classroom Interaction on Willingness to Communicate: The Mediating Roles of Speaking Self-Efficacy and Foreign Language Enjoyment*

**General Evaluation**

The manuscript addresses a timely and highly relevant topic within the field of second language acquisition (SLA), focusing on the behavioural influence of classroom interaction (CI) on students' willingness to communicate (WTC), with the mediating roles of speaking self-efficacy (SSE) and foreign language enjoyment (FLE). The topic is well-grounded in theoretical frameworks (Social Interactionist Theory and Social Cognitive Theory), and the study contributes empirical evidence to an area that is still developing. The writing is generally clear, the structure is logical, and the statistical analyses are appropriate for the proposed model.

Overall, the study is well-designed and offers meaningful contributions. However, there are several aspects where further clarification and minor revisions will significantly strengthen the manuscript, particularly in terms of methodological transparency, depth of discussion, and theoretical interpretation.

Given the solid foundation of the work and the minor nature of the revisions required, I recommend the manuscript for **Minor Revision**.

**Specific Comments**

**1. Methods**

**1.1 Sampling and Participants**

- The manuscript reports that participants were drawn from three universities in China but does not specify:
  - The types of these institutions (e.g., comprehensive universities, research-intensive, teaching-oriented, regional vs national).
  - The geographical distribution of these institutions.
  - The sampling method used (e.g., convenience sampling, random sampling, stratified sampling).

**Recommendation**:
Please explicitly describe (1) the types and regional characteristics of the sampled universities, and (2) the sampling method applied. If non-probability (e.g., convenience) sampling was used, briefly acknowledge this limitation and its potential impact on the external validity of the findings within the Limitations section. This addition will enhance the study’s transparency and provide readers with essential context for interpreting the generalisability of the results.

**1.2 Questionnaire Development and Reproducibility**

- The questionnaire was largely self-developed or adapted but the full item list is not included.
- Although the psychometric properties (reliability and validity) are reported and appear satisfactory, the absence of the full questionnaire limits the replicability of the study and prevents full scrutiny of item content.

**Recommendation**:
It is strongly advised to provide the complete questionnaire (all 44 items across four dimensions) as an appendix or supplementary material. This will allow for replication, enhance methodological rigour, and align with best practices for transparency in psychological and educational research.

**2. Discussion**

**2.1 Direct Effect vs. Mediating Effects Analysis (Section 4.1)**

- While the direct effect of CI on WTC is well-supported, the discussion would benefit from a clearer comparative analysis of the magnitude of direct vs. mediated pathways.
- The total indirect effect (0.102 + 0.171 + 0.036 = 0.309) represents a substantial portion of the total effect (total effect = 0.755), yet this proportion is not directly highlighted.

**Recommendation**:
Please deepen the discussion by quantitatively comparing the relative strengths of direct (0.447) and indirect effects (0.309). This would contribute to a richer understanding of the mechanism by which CI exerts its influence on WTC. Emphasising the prominent role of affective and cognitive mediators would advance the theoretical discussion.

**2.2 Differentiating SSE and FLE Mediation Strengths (Sections 4.2 and 4.3)**

- It is notable that FLE demonstrates a stronger mediating effect (0.171) than SSE (0.102), yet the manuscript does not attempt to interpret this difference.
- Theoretically, affective states such as enjoyment may exert a more immediate influence on communication behaviour compared to cognitive constructs like self-efficacy, particularly in anxiety-prone contexts such as foreign language learning.

**Recommendation**:
Consider incorporating a brief theoretical discussion on why FLE may have a stronger mediating effect than SSE. For example, you may draw upon Pekrun’s Control-Value Theory of Achievement Emotions, or Dewaele and MacIntyre’s research on enjoyment in language learning, to suggest that positive affect directly promotes communication willingness by mitigating anxiety and fostering engagement. This theoretical elaboration would enhance the interpretive depth of your findings.

**2.3 Potential Reciprocal Effects**

- While the manuscript appropriately applies Bandura’s concept of reciprocal determinism, the current model is unidirectional.
- Given that WTC could feasibly influence CI (e.g., more willing students might engage more in class, further enriching interaction), a brief reflection on potential bidirectional relationships would strengthen the theoretical sophistication of the paper.

**Recommendation**:
Although testing reciprocal effects is beyond the scope of this study, I suggest adding a short paragraph in the Limitations section acknowledging this issue and recommending future longitudinal or cross-lagged panel designs to explore these dynamic processes.

**Minor Points**

1. **Language and Proofreading**

- Minor typographical and grammatical errors should be corrected:
  - “boots students’ motivation” → “boosts students’ motivation”
  - “a a critical predictor” → “a critical predictor”
  - “had the right to withdrew” → “had the right to withdraw”
- A thorough proofreading is recommended prior to final submission.

2. **Consistency of Terminology**

- Ensure consistent use of terms such as "classroom interaction" rather than occasionally switching to "classroom communication".

3. **Figure Presentation**

- Figure 1, which is critical to understanding the model structure, is not included in the current version. Please ensure that all figures are properly embedded and formatted in the submission file.

**Strengths of the Study**

Despite these minor issues, the manuscript demonstrates several strengths:

- The sample size is relatively robust compared to many studies in this field.
- The model is theoretically well-justified.
- The SEM approach, combined with bootstrapping, offers rigorous empirical support.
- The study contributes valuable insights into both cognitive and affective mechanisms underlying WTC.

**Overall Recommendation: Minor Revision**

The manuscript makes a valuable contribution to the literature on language learning motivation and classroom interaction. The revisions requested are primarily clarificatory and interpretive rather than requiring substantial additional analysis. Once addressed, the manuscript will be well-positioned for publication.
